# A Digital Interface ASIC for Triple-Axis MEMS Vibratory Gyroscopes

**DOI:** 10.3390/s20195460

**Published:** 2020-09-23

**Authors:** Risheng Lv, Qiang Fu, Weiping Chen, Liang Yin, Xiaowei Liu, Yufeng Zhang

**Affiliations:** 1 MEMS Center, Harbin Institute of Technology, Harbin 150001, China; lvrisheng@hit.edu.cn (R.L.); weipingchen.hit@gmail.com (W.C.); liangyin.hit@gmail.com (L.Y.); xiaoweiliu.hit@gmail.com (X.L.); yufengzhang.hit@gmail.com (Y.Z.); 2 Key Laboratory of Micro-Systems and Micro-Structures Manufacturing (Harbin Institute of Technology), Ministry of Education, Harbin 150001, China; 3 State Key Laboratory of Urban Water Resource & Environment, Harbin Institute of Technology, Harbin 150001, China

**Keywords:** inertial navigation, MEMS vibratory gyroscopes, triple-axis digital interface ASIC, low-noise analog front end, nonlinear stabilization control, incremental zoom ADC

## Abstract

This paper proposes a solution for sensing spatial angular velocity. A high-performance digital interface application specific integrated circuit (ASIC) for triple-axis micro-electromechanical systems (MEMS) vibratory gyroscopes is presented. The technique of time multiplexing is employed for synergetic stable drive control and precise angular velocity measurement in three separate degrees of freedom (DOF). Self-excited digital closed loop drives the proof mass in sensing elements at its inherent resonant frequency for Coriolis force generation during angular rotation. The analog front ends in both drive and sense loops are comprised of low-noise charge-voltage (C/V) converters and multi-channel incremental zoom analog-to-digital converters (ADC), so that capacitance variation between combs induced by mechanical motion is transformed into digital voltage signals. Other circuitry elements, such as loop controlling and accurate demodulation modules, are all implemented in digital logics. Automatic amplitude stabilization is mainly realized by peak detection and proportion-integration (PI) control. Nonlinear digital gain adjustment is designed for rapid establishment of resonance oscillation and linearity improvement. Manufactured in a standard 0.35-μm complementary metal-oxide-semiconductor (CMOS) technology, this design achieves a bias instability of 2.1°/h and a nonlinearity of 0.012% over full-scale range.

## 1. Introduction

Recently, precise and reliable inertial navigation techniques have received significant and widespread attention in fields of both civilian and military applications. Despite accurate heading information provided by radio aids, independent navigation capacity of individual sensors is still indispensable in modern navigation systems especially when satellite service is not available [1]. Familiar examples of these circumstances include personal indoor navigation, deep-sea exploration, radio interference, and even electronic jamming during warfare. Navigation precision in such cases almost always relies on offline inertial navigation systems (INS). Inertial navigation generally involves the use of gyroscopes and accelerometers to measure instantaneous angular velocity and linear acceleration, respectively [2].

Compared with typical mechanical sensing in conventional applications of inertial navigation, MEMS gyroscopes are quite superior in some remarkable advantages including low cost, good thermal stability, high sensitivity, and simple batch fabrication and are, therefore, increasingly attractive in angular rate detection as an effective method in integrated guidance [3,4]. Besides, the employment of frequency tuning improves quality factor and reduces frequency split. Some effective and practical methods of mass adjustments, either addition or removal, have been successfully implemented for mode matching without deterioration of quality factor [5,6,7,8]. Several successful implementations have verified corresponding validity [9,10,11,12]. Especially, research on wafer-level post-fabrication frequency tuning has been developed for simultaneous reduction of the modal frequency differences, which is one of growing research potentials [13]. This process has good compatibilities with any further processing that is advantageous to batch fabrication and widespread applications of MEMS gyroscopes. After several decades of intensive development, a considerable rise in gyroscope performances has appeared thanks to innovative designs. Mode matching is one of the effective methods of reducing frequency split between drive and sense modes [14,15,16,17]. In this case, gyroscopic sensitivity depends on the resonant frequency coincidence between sense and drive modes as well as frequency selectiveness, i.e., the quality factor [18]. Besides, new gyroscopes have drawn wide focus, of which disk resonator gyroscope (DRG) is an excellent representative. The Boeing company first proposed and implemented a compact planar micro-machined design [19]. Based on the fundamental deign, many valuable explorations have also been realized including optimized ring thickness distribution and quality factor enhancement [20,21].

Technological development shed new light on the possibilities of higher integration level in both mechanical sensing elements and corresponding interface circuits. Researchers around the world have been investing abundant efforts in this field. Multiple degree-of-freedom (DOF) mechanical designs have shown various methods and possibilities to improve integration density, even by means of new materials [22,23,24,25]. On the other hand, interface integrated circuits (IC) have proven inherent advantages in minimal area, power consumption, and many other outstanding characteristics, compared with current solutions implemented by discrete devices in sensor applications. Besides, satisfying processing compatibility with MEMS manufacture also helps IC become almost exclusively mainstream in interface circuits for micromechanical sensors. Some designs of corresponding ASICs have been published in the literature for multi-axis sensing capacity [26,27,28]. These works have presented respective signal processing methods either in analog circuitry or digital alternatives, open- or closed-loop sense schemes and diverse demodulation realizations.

This work presents an original design of a triple-axis digital interface ASIC specialized for MEMS vibratory gyroscopes. The overall topological design is based on a multiplexed zoom analog-to-digital converter (ADC). The implemented ADC employs an incremental principle, combining successive approximation register (SAR) and ΣΔ architectures. Cooperating with high-accuracy sampling holders (S/Hs), this ADC allows synchronous multiple analog voltage inputs and achieves precise digitalized conversion. Based on the employed technique of multiplexing data conversion, the proposed interface ASIC avoids redundant design and obtains capacity of triple-axis angular velocity sensing. Rest digital controlling logics and resolving circuits are shared among axes. In other words, mechanical motion control and electrical signal processing of the three orthogonality assembled gyroscopes are performed in one single ASIC. Additionally, this also helps eliminate superfluous coupling interferences. Relevant signals in both drive and sense loops are initially converted into digital forms and are ready to be processed by latter digital circuit modules. Such processing pattern is readily able to provide digital outputs naturally to match dominant digital controlling systems in modern industrial or consumer products. Additionally, digital signals have inherent robustness to many environmental changes and electromagnetic interference (EMI). This is also favorable for anti-interference capability and insensitivity to temperature drift. On the foundation of precise data conversion, signal processing is accomplished in digital domain. Therefore, more flexible algorithms can be realized in contrast to restricted methods in analog circuits. A digital proportion-integration (PI) controller is employed in drive loop for maintaining stability and automatic control. In respect to mechanical activation of drive loop, nonlinear gain adjustment is designed to balance between rapid oscillation and accurate driving control.

This paper is organized as follows. Section 2 illustrates the mechanical vibration model. System overview and implementation details are described in Section 3 and Section 4, respectively. Experimental results are shown in Section 5 and discussed in Section 6. Comprehensive conclusions are finally drawn in Section 7.

## 2. Mechanical Sensing Element

**Figure 1:**
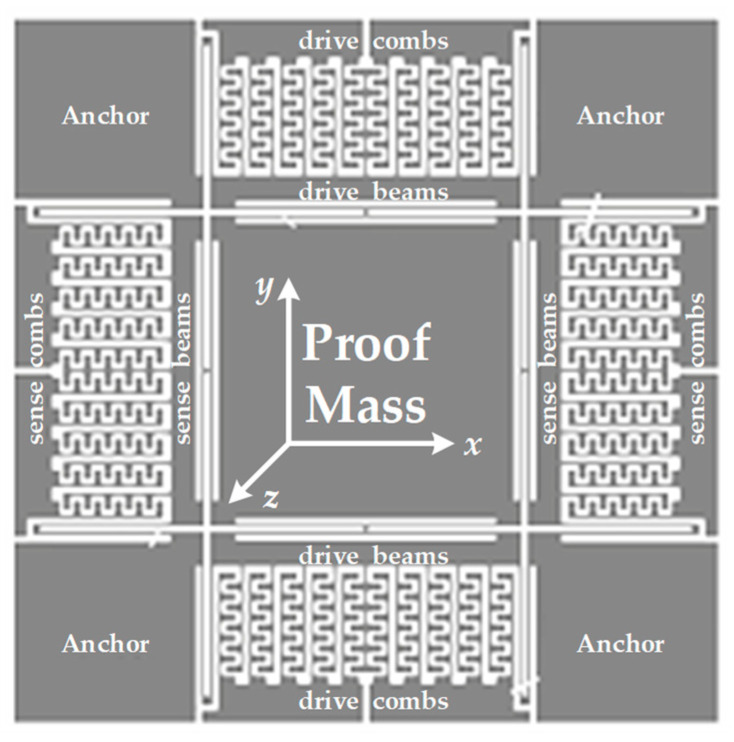
A standard model of the sensitive structure for micro-electromechanical systems (MEMS) gyroscopes.

Common MEMS vibratory gyroscopes are composed generally of a proof mass with elastic beams and connecting mechanical combs in variable equivalent capacitors. MEMS sensing elements are usually packaged in vacuum cavities for lower Brawnian noise according to actual application requirements. Mechanical comb structures include fixed and free combs in both drive and sense directions. All fixed combs are directly connected to the substrate, and no relative motion is allowed. Four anchors are equably set in ambient corners and connect free combs in drive and sense axes via surrounding elastic beams. Additionally, the central proof mass is connected to free combs in both axes by elastic beams and motions in the planar plane. These fundamental structural units describe mechanical parametric variation based on Coriolis effect. A representative model of holosymmetric mechanical structures for MEMS vibratory gyroscopes with a central single proof mass is shown in Figure 1. An ideal situation of stable electrostatic driving means a moderate resonance oscillation of the proof mass in the direction of *x*-axis, which is one of the necessary conditions of the Coriolis effect. Specifically, an external angular velocity input in *z*-axis activates an orthometric Coriolis force in *y*-axis. In consequence, there is a corresponding forced vibration in *y*-axis resulting from introduced Coriolis force.

**Figure 2:**
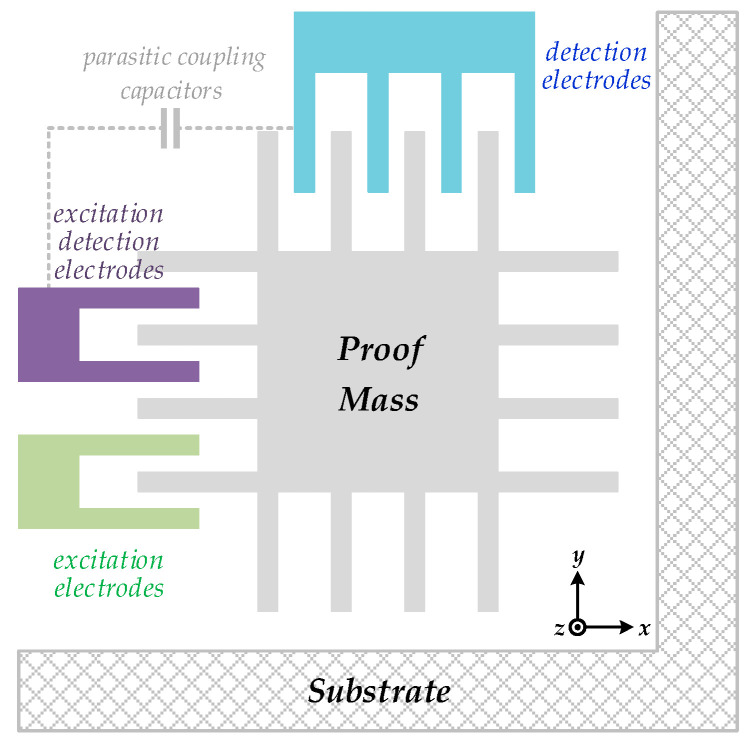
A simplified model of the sense element of MEMS vibratory gyroscopes.

The sensitive orientation is, therefore, vertical to the manufactured plane, namely, the *z*-axis. This gyroscope employs a symmetrical design, in which the vibratory proof mass is in the center and four anchors in the corners are designed for fixation. Both drive and sense combs are connected indirectly to the central proof mass through each internal elastic beam. Figure 2 plots a simplified model of the sense element of MEMS vibratory gyroscopes. Sensitive capacitors between these combs vary with real-time state of mechanical motion. This capacitance variation is detected by corresponding interface circuits, which always begin with C/V conversion. The drive resonator is embedded in a closed drive loop to sustain constant oscillation at its inherent resonance frequency for Coriolis force generation. The employed MEMS gyroscope is fabricated in silicon-on-insulator (SOI) process using a notching effect to release the mechanical structure [29]. Besides, a high-vacuum packaging is also adopted to suppress bias sources by increasing the quality factor (Q) of the overall sense element [30].

Identical sense elements are employed in triple axes for maximum consistency of mechanical parameters, except for possible minor manufacturing errors among triple axes. Actual design properties are listed in Table 1, including quality factors, resonance frequencies, etc.

In drive mode, stable resonance is sustained by a pair of differential alternating voltages loaded on capacitors between driving combs. Resultant electrostatic forces drive the proof mass into vibration. When this driving force shares the same frequency with mechanical natural frequency, maximum sensitivity is obtained in the overall gyroscope system. In the mechanical structure of MEMS gyroscope sense elements, capacitance between a pair of fixed and free combs is calculated as

(1) $$C=2\varepsilon \frac{z\cdot x}{y}$$

where *ε* denotes dielectric constant. The actual area of double electrodes of this variable capacitor is z·x and the electrode interval is *y*. When the proof mass is in periodic mechanical vibration, capacitance varies correspondingly. Only vibration displacement *x* is affected in drive axis, so that the consequent electrostatic force between combs is, therefore, expressed as

(2) $$\vec{F}=\frac{1}{2}\nabla C(x,y,z)V^{2}=\frac{1}{2}\frac{\partial C}{\partial x}V^{2}=\varepsilon \frac{z}{y}V^{2}$$

where *V* is the loaded voltage on this variable capacitor. If a couple of driving voltages follow $V_{dc}\pm V_{ac}\sin\omega t$, the resultant electrostatic force on driving capacitor is calculated as

(3) $$\left|\vec{F}\right|=N\varepsilon \frac{z}{y}\left[{\left(V_{dc}+V_{ac}\sin\omega t\right)}^{2}-{\left(V_{dc}-V_{ac}\sin\omega t\right)}^{2}\right]=4N\varepsilon \frac{z}{y}V_{dc}\cdot V_{ac}\sin\omega t=F_{0}\sin\omega t$$

where *N* is the number of driving combs. Obviously, the resultant driving force has the same frequency component with external driving voltage.

According to dynamic characteristics in mechanical structure, physical motion in *x*-axis of the proof mass is described as

(4) $$x(t)=B_{d}\sin(\omega t+\varphi_{d})$$

where *B_d_* denotes the vibration amplitude. When driving frequency ω equals the inherent natural frequency of the default mechanical structure, *φ_d_* is calculated as −π/2. Generally, the input angular velocity Ω in *z*-axis has a relatively low frequency of $\omega_{i}$ and an amplitude of $\Omega_{0}$. We can simply denote it as

(5) $$\Omega =\Omega_{0}\cos\omega_{i}t.$$

On the other hand, the inherent resonance frequency in *y*-axis vibration is demodulated to double frequency and is filtered afterwards. As a result, frequency and amplitude of input angular velocity remain in low-frequency signal band. Demodulated motional oscillation $y^{\prime }(t)$ in *y*-axis can be expressed as

(6) $$y^{\prime }(t)=B_{s}\cos(\omega_{i}t+\varphi_{s}).$$

Obviously, the vibration pattern in *y*-axis carries complete information of the input angular velocity, which can be accurately resolved accordingly.

## 3. System Overview and Topology Analysis

**Figure 3:**
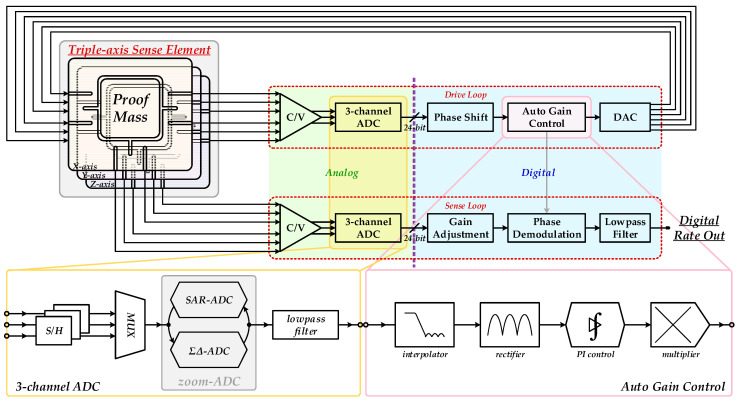
Systematic design of the proposed interface ASIC for MEMS gyroscopes.

A fine interface ASIC performs precise mechanical control, electrical signal detection, and processing. The proposed interface ASIC for triple-axis MEMS vibratory gyroscopes is mainly composed of a self-excited closed drive loop as well as an open loop for angular velocity sensing, as shown in Figure 3. Specifically, the drive loop realizes stable self-excited vibration and resonance oscillation. Sense loop responds to and extracts from motional variation in mechanical structures and resolves initial input angular velocity as the final output. Both loops begin with analog front-end circuit modules composed of accurate C/V conversion and triple-channel high-precision data conversion. Rest circuit elements are all implemented as digital modules.

Either drive or sense loop begins with low-noise C/V converters to gain an electrical voltage signal reflecting mechanically motional properties from charge storage in variable capacitors between free mechanical combs. This module communicates mechanical and electrical domains in MEMS gyroscopes and is, therefore, a key unit in overall interface ASIC. Analog output voltages of C/V converters carry physical information of mechanical vibration and can be properly processed by latter circuitry system. Signal transition from analog to digital forms is implemented by a triple-channel incremental zoom ADC using the technique of time-sharing multiplexing. The employed ADC combines SAR and ΣΔ patterns and, therefore, reconciles the key indexes of conversion speed and accuracy, which are the main advantages in both converters, respectively. Input signals are processed by SAR and ΣΔ conversion in sequence. Triple voltage signals from the C/V converter are first sampled by S/Hs. Circuit resources in this ADC are uniformly managed by a multiplexer (MUX) via global timing sequence. Final digital output is achieved after low-pass filtering which cancels high-frequency modulated noise components in ΣΔ modulation to gain a flat passband. Specifically, parallel data streams are received synchronously and converted successively into equivalent digital signals by this ADC. The operating pattern of synchronous sampling also helps minimize phase mismatch between triple channels as well as both drive and sense modes so that deterioration effects resulting from consequent phase noises on overall gyroscope performances are suppressed effectively. This ADC enables the characteristics of triple signal processing in overall gyroscope interface ASIC. All circuit modules in correspondence with digital signal processing and angular velocity resolving in both drive and sense loops are multiplexed. In this way, mechanical sense elements in triple orthogonal gyroscope axes are effectively driven and controlled to achieve angular velocity detection in spatial orientations. Furthermore, device economization and energy efficiency are additionally achieved since repetitive interface circuits in triple gyroscope axes are integrated as multiplexed mode and redundant circuit elements are avoided.

Besides, the ability of receiving simultaneous inputs also enables a multiplexed interface ASIC. Rest circuit modules are designed in digital logics to realize better compatibility and higher integration. This is, additionally, also beneficial to systematic capacity of resistance to unexpected interference in actual working conditions, such as electromagnetic interference.

### 3.1. Self-Excited Drive Loop

The constant and stable resonance oscillation comes from a self-excited period since electrification. Vast white noise in wide band gets through a preliminary selection by mechanical properties. Sensing elements have inherent natural frequencies and provide frequency-selection characteristic, filtering most noise ingredients except for only a specific frequency. Owing to the property of loop positive feedback, the targeted signal at this single frequency is enhanced over and over while others are suppressed substantially. Moreover, the basic principle of Barkhausen’s criteria is obligatory, which can be expressed as

(7) $$\left\{ \begin{array}{l} \left|\beta H(j\omega_{0})\right|=1\\ ∠\beta H(j\omega_{0})=-180^{\circ } \end{array}\right..$$

We use *β* and *H*(*s*) here to denote feedback gain and open-loop transfer function, respectively. It is evidently important that correct and accurate phase relation is ensured. Nevertheless, the closed drive loop includes both mechanical and electrical elements, most of which bring diverse phase delay. In consequence, precise phase control and adjustment are significant. In other words, tiny phase deviation may be fatal to overall systematic sensitivity and noise level. For a brief illustration, mechanical and electrical influences are analyzed in sequence.

On the aspect of mechanical structures, phase change induced by transfer characteristics can be seen in dynamic calculation. According to Equations (3) and (4), an obvious phase delay occurs in the sensing element. In drive mode, output differential resonance signals show a 90° delay from input alternating driving voltages. This phase lag should be compensated in time to guarantee an effective self-excited loop.

Besides, functional circuit modules also introduce relative phase variation. For example, the modules of auto gain control and filters include many digital logics implemented by numerous integrators and differentiators and, thus, result in phase delay. Apart from digital components, analog modulators have similar effects as well. So, precise phase shift is essential and crucial.

Start-up time is also a major concern in some specific applications. Generally, proof mass is driven by square-wave or sinusoidal signals. Both methods, however, have their own drawbacks. Square driving waves mix much superfluous noise due to intrinsically complicated frequency constituents. In comparison, sinusoidal driving signals cannot provide adequate initial gain and, consequently, lead to a prolonged period of resonance establishment [31]. This work employed an improved sinusoidal-driving scheme by nonlinear gain adjustment in digital domain to balance between rapid oscillation and resonance accuracy. An adequate gain is adopted at first so that rapid and low-distortion driving is achieved. The whole system then trends to a dynamic and stable balance gradually. Complementary gain reduces as oscillation forms and, finally, maintains the unit after stabilization.

### 3.2. Precise Sense Loop

As illustrated above, drive mode has almost no alternative except for a closed-loop design in order to sustain a stable and accurate state of resonance. Compared with an almost exclusive choice of drive loop, either an open or closed loop has unique advantages over the other [28]. Generally, a closed sense loop cancels mechanical vibration in the direction of Coriolis force and obtains a relatively static condition. However, mode matching between drive and sense modes is usually obligatory. Minor phase deviation, consequently, leads to a radical gain attenuation, in which case incredible performance deterioration occurs. On the other hand, the proof mass remains in mechanical vibration in an open sense loop. Motion-induced differential capacitance variation is detected by analog front end in sense circuit, namely, C/V converter. Inherent stability and robustness make the topology of open sense loop quite effective and advantageous in many applications. Based on the above considerations, this work concentrated on the long-term performance reliability and, therefore, employed an optimized open sense loop.

Precise resolving of initial input angular velocity is one of the most significant functions in sense loop. Sense signals carry necessary motional characteristics yet still need further processing. The stable resonance signal in drive loop is also introduced as demodulation reference. This work applied the method of phase demodulation to avoid massive multiplier utilization occupying large chip areas. Despite low-pass filtering impacts in former ADC, there still remain residual high-frequency noises from demodulation in sense loop. After removal of these interferences by a low-pass filter, a final digital output is obtained for representation of initial input angular velocity. After a further compensation algorithm, better gyroscope performances are achieved.

### 3.3. Multi-Channel Zoom ADC

Representative data conversion in zoom mode is usually implemented by synergetic hybrid ADCs, which are divided as coarse converters and fine equivalents [32]. A six-bit SAR ADC and a third-order typical cascade-of-integrators and feedforward form (CIFF) ΣΔ ADC were employed in this work for advance coarse conversion and subsequent accurate computation. As the SAR conversion is mainly executed by feedback capacitor array in successive cycles, such design is advantageous to an efficient balance between conversion accuracy and chip area. As a result, the two types of converters perform alternately in a whole conversion cycle. A standard SAR ADC is outstanding in the aspect of conversion speed and power efficiency, while high precision is characteristic of a ΣΔ ADC [33,34,35,36]. The implemented zoom conversion in this work united both advantages and was, therefore, more efficient, so that rigorous design requirements for both ADC components of the above two types were no longer necessary. In consequence, there was more optimization space for power economization and chip area. Specifically, prompt and approximate estimation was first realized in the initial six clock periods by coarse SAR converter, which is referred to as dynamic feedback in subsequent fine conversion and final most significant bits (MSBs). In this way, most quantization errors in ΣΔ phase are excluded in advance. Residual errors are highly suppressed and restricted within a definite range, depending on the effective bits of the coarse conversion [37]. A single-bit quantizer, therefore, is enough for effective suppression of most quantization noises in contrast to a standard ΣΔ modulator. Apart from the obvious reduction of circuit complexity, satisfactory conversion linearity is inherent in single-bit quantization.

Supposing a six-bit SAR conversion, single quantization interval is a 1/2^6^ portion of supply voltage range. Smaller residual input voltages of ΣΔ conversion further decrease overload possibilities and improve compatibility with stronger noise shaping implemented by high-order modulation. Moreover, a moderate oversampling ratio (OSR) is adequate and incidentally contributes to lower power dissipation and tolerant design indexes for key circuit modules.

**Figure 4:**
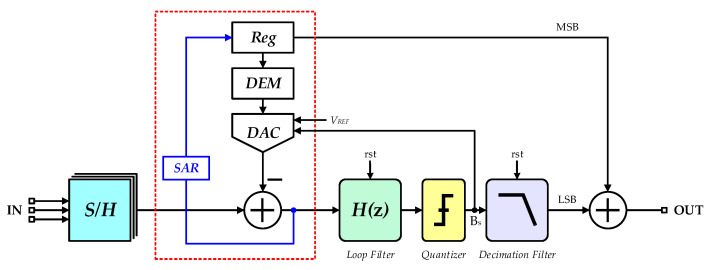
Block diagram of the proposed incremental zoom ADC.

Figure 4 plots the overall system architecture of the proposed incremental zoom ADC. Based on the above illustration, coarse results of SAR conversion are regarded as the MSBs of the final digital output. Besides, employed voltage references in ΣΔ conversion were also fixed accordingly. The employed reference range in this work was double the least significant bits (LSBs) of SAR converter and was refreshed in the next conversion cycle. Hence, general input swings of ΣΔ modulator were totally covered by dynamically adjusted references. The LSBs of the final output were calculated from ΣΔ conversion. The final digital output, *D_out_*, was derived from combining the two conversion steps and is expressed as

(8) $$D_{out}=D_{SAR}\cdot 2^{n-1}+D_{\Sigma \Delta }$$

where *D_SAR_* and *D*_ΣΔ_ are conversion outputs of the two steps, respectively. Bit resolution of fine ΣΔ converter is denoted as *n*.

Accurate initial processing of multiple input voltages by S/Hs is a fundamental prerequisite for precise multi-channel data conversion realized by the presented zoom ADC. Quiet and precise S/Hs are one of the core determinants of overall conversion precision. Either unreasonable noise density or excessive harmonic distortion leads to inevitable deterioration in data fidelity of signal conversion. Final accuracy depends on performances of the inferior one between preceding S/Hs and subsequent zoom ADC, which constitute an entire multiplexed incremental zoom ADC together.

**Figure 5:**
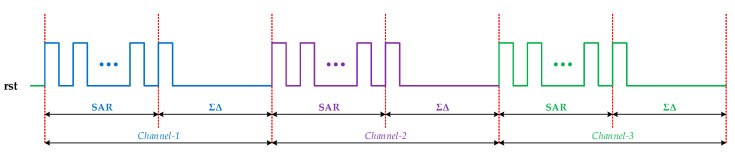
Timing diagram of incremental zoom ADC.

The data conversion of the incremental design scheme is mainly controlled by a reset signal *rst* at the beginning of each conversion period. This reset signal enables the loop filter in analog modulator and digital decimation filter to return to initial state and to get rid of memory status in integrator circuits. Residual errors in former data conversion, in consequence, are effectively eliminated and will not participate in successive calculations. The overall timing design of the reset signal is shown in Figure 5. Based on the multiplexed design in this work, analog voltage signals in each channel were converted to digital forms in an entire cycle of sequential SAR and ΣΔ conversion. Besides, multiple inputs were controlled by global timing and digitalized by periodic process so that multi-channel and multiplexed digital conversion was accomplished.

## 4. Circuit Implementation Details

Since most controlling and resolving algorithms were realized by digital logics in this work, circuitry design gives priority to analog modules in this section. Most analog circuit components consist of the front-end stage, including high-precision C/V converter and the incremental zoom ADC mentioned above. In addition to some digital controlling logics and filters, the presented ADC was mainly composed of differential sampling holders and analog modulators.

### 4.1. High-Precision C/V Converter

**Figure 6:**
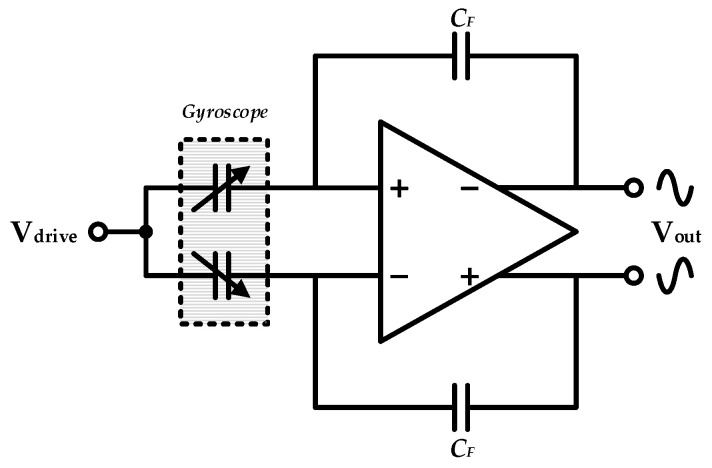
Differential C/V converter in the proposed interface ASIC.

The implemented drive loop consisted mainly of a differential C/V conversion module for capacitance detection and a section of stability control. Specifically, variable capacitors between mechanical combs and analog front-end stage circuit (operational amplifier and feedback capacitors) composed the differential charge amplifier for C/V conversion, as shown in Figure 6.

The following module is a digital amplitude controlling segment based on a multi-channel ADC. Auto gain control is comprised of rectifying low-pass filtering and nonlinear gain adjustment. A multiplexed digital-to-analog converter (DAC) concludes the closed drive loop, whose analog outputs are feedbacked to corresponding channels to accomplish entire servo control and electrostatic actuation.

### 4.2. Differential Sampling Holder

**Figure 7:**
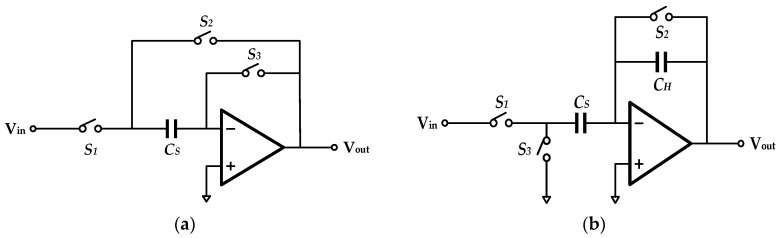
(**a**) Flip-around sampling holder, (**b**) charge-redistribution sampling holder.

Typical switched-capacitor (SC) S/Hs commonly include two specific realizations, namely, flip-around and charge redistribution, as shown in Figure 7a,b. The most obvious difference lies in the pattern of charge distribution. Flip-around S/Hs employ a shared capacitor between sampling and holding periods, while charge-redistribution S/Hs need two separate capacitors for charge storage. Entire signal processing includes two states in both S/Hs, which are sampling phase and holding phase. The actual circuit status is dependent on switches controlled by a double-phase clock.

The main difference between both S/Hs exists in patterns of charge storage. Charge-redistribution S/Hs sample input voltages and store corresponding charges on sampling capacitor *C_S_*. These charges are redistributed during the following holding phase. It is noted that the sampling capacitor *C_S_* still reverses partial charges. This means the holding capacitor *C_H_* cannot collect all charges. On the contrary, flip-around S/Hs contain no holding capacitor. A single capacitor is shared between sampling and holding phases. If parasitic capacitance is omitted, flip-around S/Hs have a larger feedback ratio. Therefore, design requirements for gain-bandwidth (GBW) product are quite relaxed. Furthermore, power consumption reduces correspondingly.

Specifically, input noise voltage in both circuits is identical and calculated as

(9) $$v_{n}^{2}=\frac{8\pi kT}{3\beta C_{L}}$$

where *k* is the Boltzmann’s constant and *β* and *C_L_* denote feedback ratio and load capacitance, respectively. Flip-around S/Hs have lower noise voltage due to larger feedback ratio. Additionally, shared capacitor means less layout area, which immediately decreases circuit cost.

**Figure 8:**
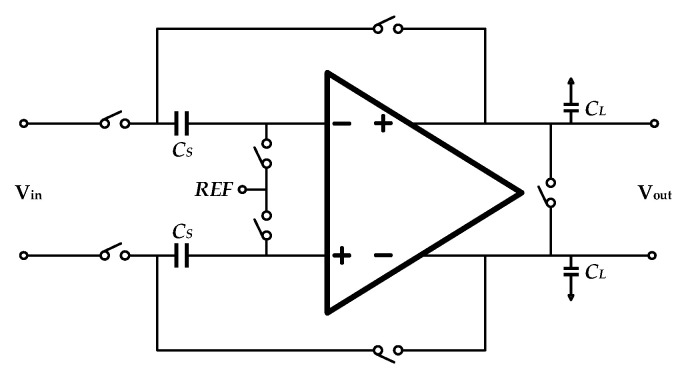
Overall design of the employed differential S/H.

Based on the above circuit analysis and noise calculation, a differential flip-around S/H (in Figure 8) was employed in this work. The differential design is advantageous to even-order distortion elimination.

**Figure 9:**
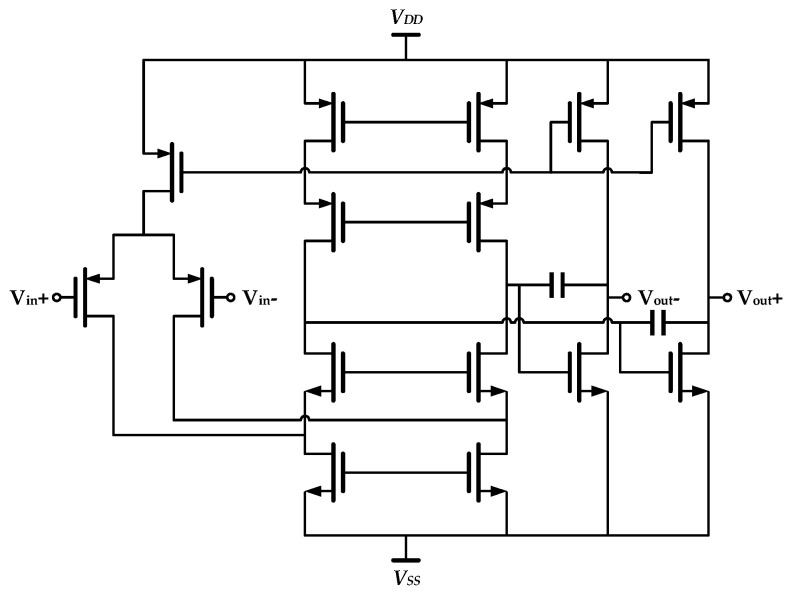
Schematic of OTA in the employed differential S/H.

During the holding phase of S/H, there are nonideal errors in output voltages resulting from finite DC gain of the core amplifier. This will lead to corresponding precision loss. As a whole circuit module, overall conversion accuracy is limited by the worst element in the entire multi-channel ADC. In order to take full advantage of conversion precision in zoom ADC, output errors in S/Hs are restricted accordingly. In other words, output precision of S/H should exceed the overall precision in zoom conversion. The DC characteristic in the core amplifier is, therefore, significant. In this work, a second-order differential folded-cascade operational transconductance amplifier (OTA) (in Figure 9) was employed to realize the demanded requirements for high DC gain, gain-bandwidth (GBW) product and slew rate.

### 4.3. Analog Modulator Design

Compared with typical SAR conversion, mainly implemented by proportional capacitor array, a third-order cascaded integrator modulation was shared between both SAR and ΣΔ conversion period in this work. The whole conversion process was constructed by consecutive SAR and ΣΔ conversion. The core element of the presented zoom converter was noise-shaping modulation implemented by an analog modulator. This was essentially a specialized circuit element for noise processing, which moved in-band noises to high frequency by oversampling and characteristics of transfer function. Therefore, this modulator presented lowpass properties.

**Figure 10:**
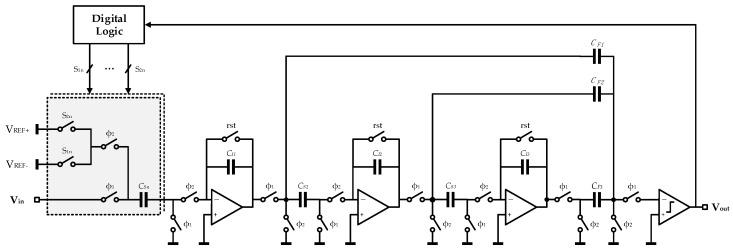
Simplified schematic of the implemented modulator.

According to the above analysis about the overall modulation system, the employed analog modulator was designed in fully differential topology for its unique superiority in common-mode noise suppression, power noise rejection, and even-order distortion elimination over single-ended architectures. The employed differential topology, additionally, introduced an extra 3 dB higher dynamic range (DR). Figure 10 plots a simplified schematic diagram for clarity. A 64-unit capacitor array acts as a feedback DAC to achieve six-bit coarse SAR conversion in advance of precise ΣΔ process. During a single SAR period, switches S_1n_~S_2n_ in feedback array are controlled by digital logic and are correspondingly connected to either V_REF+_ or V_REF−_, which dominate dynamic reference voltages. The reference range shrinks gradually with SAR conversion cycles. The required feedback linearity depends on this feedback DAC and is improved by calibration technique of data weighted averaging (DWA), one of the common methods of dynamic element matching (DEM).

After six SAR conversion cycles, a minimum reference range covering residual inputs of the following ΣΔ conversion was finally achieved. Once the input channel switched over, all integrators were reset to cancel the previous charge status in case of integration memory effect. Therefore, the implemented hybrid zoom ADC was able to convert multi-channel inputs.

Parasitic-insensitive integrators were adopted in the cascade loop for suppressing charge injection in CMOS switches with resulting precision loss. A passive adder comprised of SC paths was employed here to sum up outputs from all integrators, since it had a higher ratio of resolution to power consumption over an active adder.

**Figure 11:**
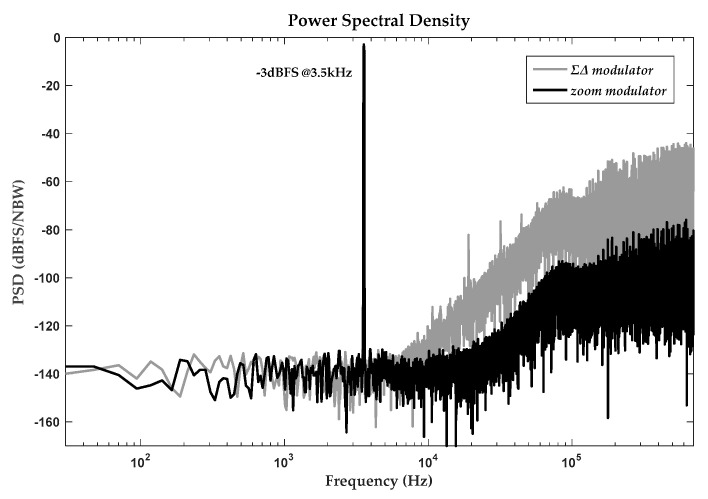
PSD comparison of ΣΔ and zoom modulator.

Overall conversion accuracy of the above modulator depended on in-band white noise, which almost always comes from circuit elements, especially in the first integrator. When advance coarse SAR conversion was bypassed, this presented modulator could also perform in conventional ΣΔ conversion. It was noted that systematic transfer and filtering characteristics differed between ΣΔ and zoom patterns, even in the condition of identical system coefficients and circuit modules. The simulated power spectral density (PSD) of double patterns are plotted in Figure 11 together for clear caparison. Generally, there is hardly any difference in noise floors of modulators since in-band noise densities are decided by white noise, as illustrated above. The obtained transfer characteristics concern subsequent digital decimation filter. If the passband of the decimation filter is designed at ΣΔ bandwidth, zoom modulator has a wider signal passband. On the contrary, if this passband is designed at zoom bandwidth, partial residual noise components of ΣΔ modulator remain in overall bandwidth and result in accuracy deterioration. In other words, quantization noises in ΣΔ modulator exceed those in zoom modulator. This is also derivable from the obviously higher out-band noise energy in ΣΔ modulator. Besides, harmonic distortion and interferences in zoom modulator are also relatively lower due to the narrower reference range provided by primary SAR conversion.

**Figure 12:**
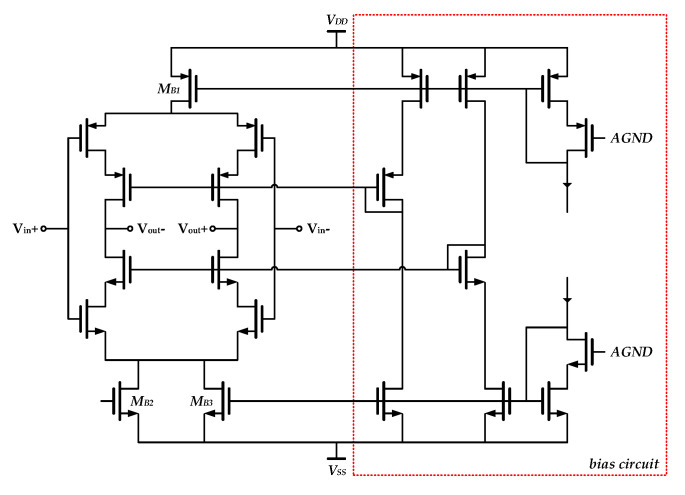
Schematic of inverter-based OTA in analog modulator.

The employed zoom operation and dynamic adjustment of reference voltage ranges resulted in a sharp fall in the equivalent input swing of the analog modulator in this work. This voltage swing was totally restricted in a range of double LSBs of the coarse SAR conversion (2V_LSB,SAR_). The practical reference voltage range was rechecked and updated before the next conversion period to cover residual input signals. Therefore, the setup process of integration signals in cascade integrators could be relatively relaxed, which also avoided extremely rigorous performance indexes for circuit modules, especially in gain and slew rate of amplifiers. An inverter-based OTA was employed to improve energy efficiency and save power dissipation, as shown in Figure 12. Specifically, the transistors M_B1_, M_B2_, and M_B3_ provided static current and embedded cascode structure enhanced DC gain.

**Figure 13:**
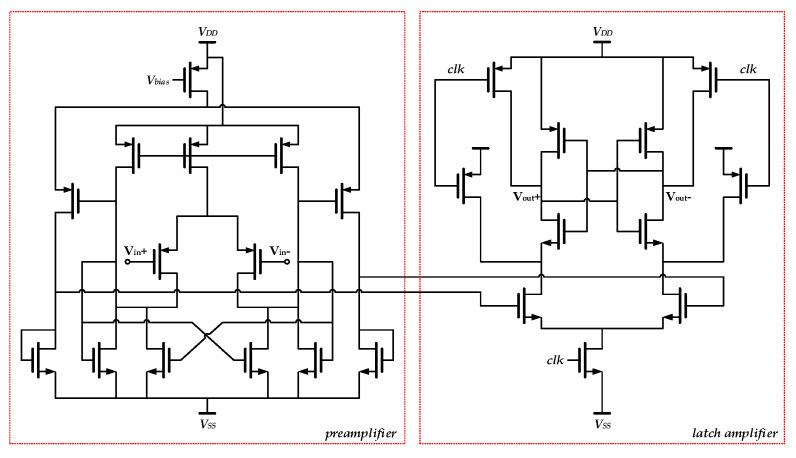
Schematic of dynamic latch quantizer in analog modulator.

Differential reference voltage ranges are constantly adjusted to cover input signal swings in zoom conversion. As a result, quantization errors in modulation loop are adequately suppressed. Moreover, this restricted amplitude also decreases gradually in the chain of cascade integrators due to the convergence properties of system parameters in modulation systems. In this case, input voltage amplitudes of quantizer were relatively small. Requirements for resolution capacity of employed quantizer, therefore, increased largely for accurate quantization. The implemented dynamic latch quantizer in this design included pre-amplification function and was composed of a pre-amplifier and a latch amplifier, so that resolution properties of rapid response and high accuracy. When a tiny voltage inputs, extra gain was introduced by the pre-amplifier. Final quantization result was output by latch amplifier. The whole circuit architecture of quantizer in this design is shown in Figure 13. Simulation results showed resolution capacity of 1 mV and output delay of 1ns.

## 5. Experimental Results

**Figure 14:**
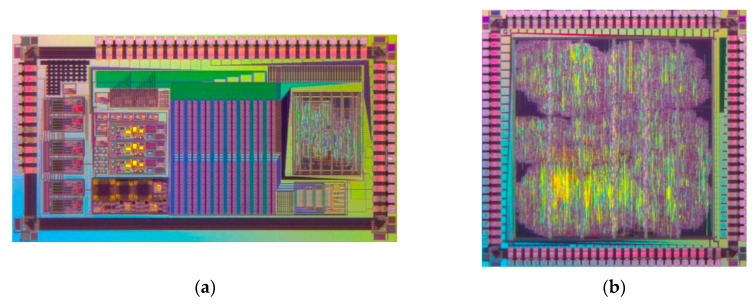
(**a**) Chip micrograph of analog modules; (**b**) Chip micrograph of digital modules.

The proposed triple-axis digital interface ASIC for MEMS vibratory gyroscopes was designed and fabricated in a standard 0.35-μm CMOS technology. Regrettably, this work did not accomplish a monolithic design by virtue of technical restraint in manufacturing process. The implemented biplate scheme divided the whole interface by analog and digital circuit elements into two chips. Specifically, analog C/V converters and multiplexed ADCs in both loops were integrated in one single chip and all rest digital modules in the other. The chip micrographs are shown in Figure 14, occupying active areas of 5.1 mm^2^ and 4.2 mm^2^, respectively. In spite of the less satisfactory integration, involuntary physical isolation between analog and digital devices resulted in compulsive interference repression. Moreover, a separate supply system was used in analog and digital blocks for coupling suppression and nonideality elimination.

**Figure 15:**
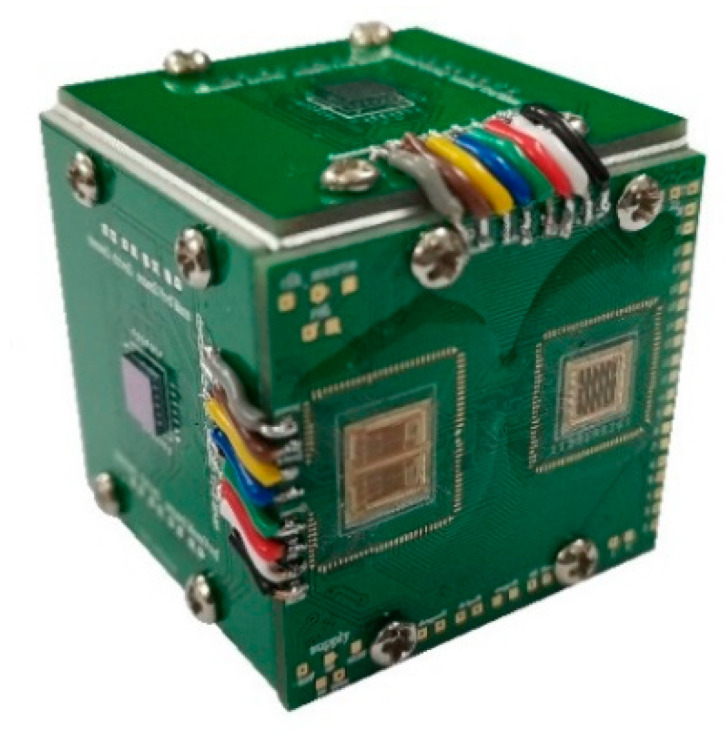
The implemented prototype gyroscope.

Figure 15 gives a general idea of the prototype integrated device consisting of three single-axis gyroscope sensing elements and the proposed interface ASIC in this work. Specifically, these three sensing elements were assembled orthogonally with each other in response to input angular velocity in respective axis. Entire circuit modules in interface ASIC were implemented by double chips, as shown in Figure 14. The double chips were bonded by metal wire generally composed of silicon and aluminum on a separate printed circuit board (PCB), which also contained a DC power supply system. All electrodes of micromechanical gyroscope structures were connected to this PCB for signal and power transmission. In addition, application of digital integrated circuits avoided most of the discrete elements (off-chip resistors, capacitors, etc.) in analog interface designs and, incidentally, improved integration.

The overall performance in an entire gyroscope system is closely related to noise level, which contains both mechanical and electrical noise induced by the sensing element and corresponding interface ASIC, respectively. In order to demarcate noise performances, ordinal measurement is necessary. The main noise source in a sense loop is nothing else than front-end C/V converter, multiplexed ADC, and terminal digital filter. Individual performance assessment makes for rational device matching and avoids over- or under-design, and, thus, leads to a balanced and optimized comprehensive properties.

**Figure 16:**
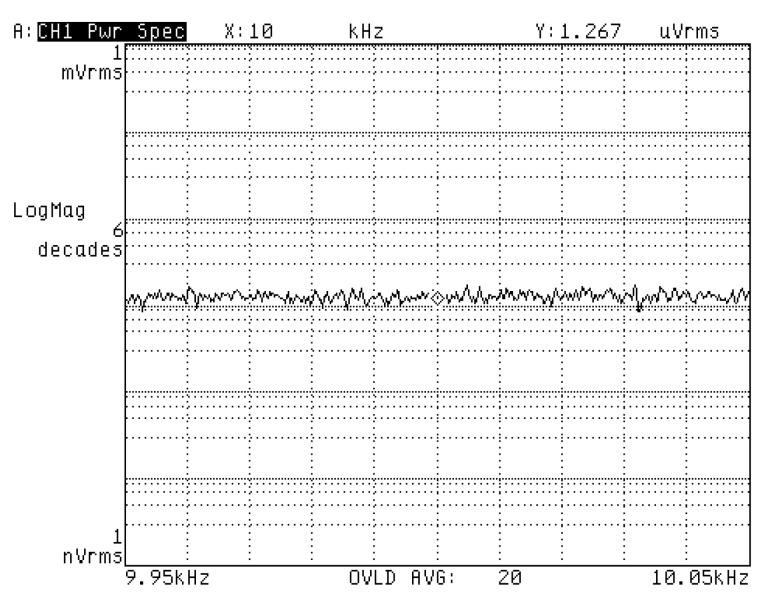
Output noise of the implemented C/V converter.

The implemented ASIC began with a differential C/V converter in both drive and sense loops, as shown in Figure 3. This conversion accomplished a key transition from mechanical motion to electrical signals. As a result, conversion accuracy had almost decisive implications on systematic noise level. It is also essential to note that this formed one of the critical noise components in the entire MEMS system. Experimental results showed voltage noise density after C/V conversion was measured as 1.267 μVrms/Hz. The actual output power spectrum by a dynamic analyzer is shown in Figure 16. The white noise level was related to low-frequency deviation in analysis of root Allan variance.

**Figure 17:**
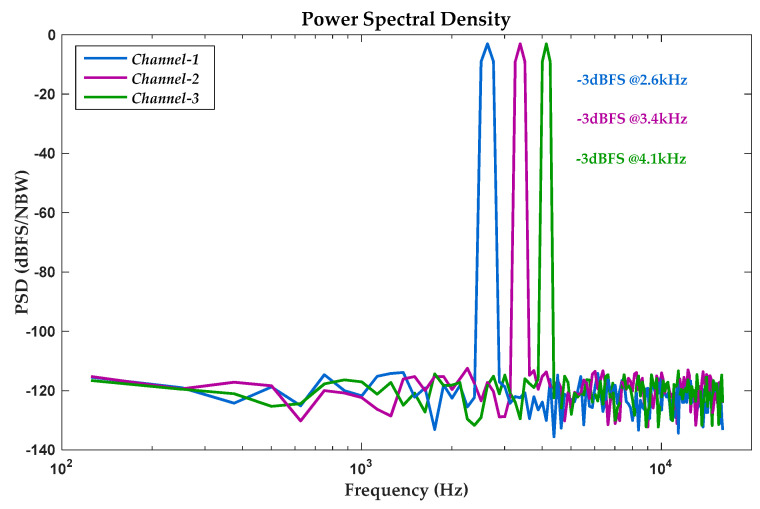
Measured output PSD of triple-channel ADC on 256 samples.

Three simultaneous disparate −3dBFS parallel inputs were selected as test signals of the prototype ADC, whose frequencies were 2.6, 3.4, and 4.1 kHz, respectively. This multiplexed zoom ADC was tested under a master clock of 15.36MHz. The measured output PSD of the implemented ADC is shown in Figure 17. As a result of the systemically embedded digital decimator, an even passband within 16 kHz was achieved in all channels, which covered most typical resonance frequencies of MEMS vibratory gyroscopes [38]. Unexpected noises (e.g., idle tones) were nearly eliminated within the signal band, while only slight low-frequency environmental interferences still remained. Experimental results proved consistent performances among triple channels, corresponding to an equivalent effective number of bits (ENOBs) over 16bits. Calculated signal-noise-ratio (SNR) of triple channels were 100.2, 99.6, and 99.8 dB, respectively.

**Figure 18:**
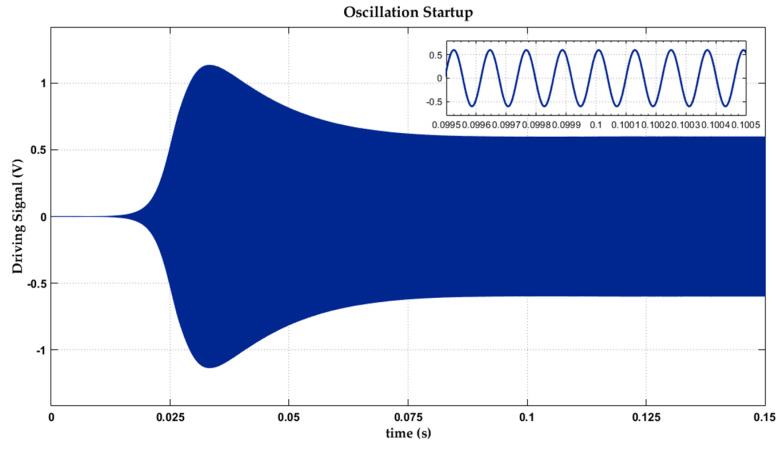
Start-up process of the closed drive loop.

Stabilized and sustaining resonance is a basic condition of the Coriolis effect. A pure oscillation excluding distortion components ensures remarkable sensing capacity in a high-precision gyroscope. Much frequency disturbance induces undesired noises and results in unknown precision loss. In some particularly rigorous applications, start-up time is also one of the core indicators. The start-up process of self-activated closed drive loop is shown in Figure 18, revealing a standard PI control course. The nonlinear gain adjustment period is clarified as well. An initial huge gain is injected for a rapid oscillation. After establishment of mechanical resonance vibration, extra gain reduces gradually back to unit. The complete oscillation process is driven by sinusoidal electrostatic excitation signals at mechanical intrinsic frequency with only amplitude modulation. Figure 18 shows a stable resonance start-up within 150 ms. The implemented sinusoidal driving degrades most distortion.

**Figure 19:**
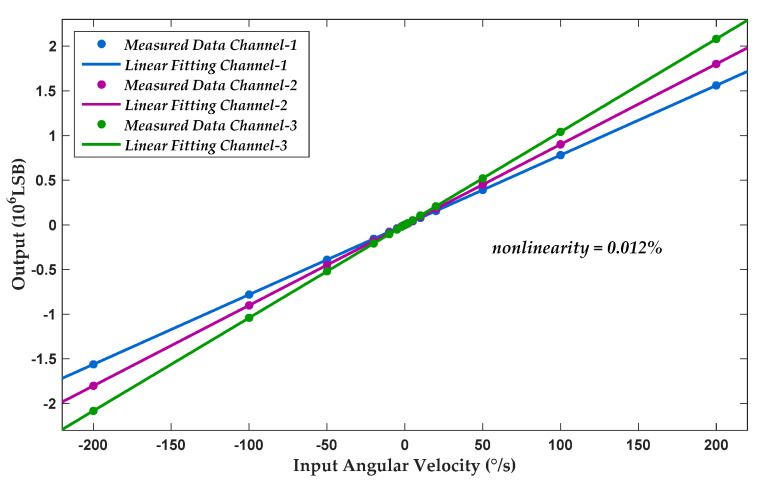
Measured DC transfer function.

The measured DC transfer characteristics of the gyroscope are presented in Figure 19. Complete output features of triple axes are shown in a single coordinate system for intuitive comparison. It shows minor diversities in scale factors among different axes. For representative test results, 23 typical measurement inputs were selected to cover full sensitivity range in each channel. At each point, three repetitive tests were carried out, and the average result was recorded as the final output. Based on the summarized measurement data, linear fitting was calculated. Under the condition of an input dynamic range of ±200°/s, experimental results showed a maximum fitting nonlinearity of 0.012% over the full-scale input range in a bandwidth of 100 Hz.

**Figure 20:**
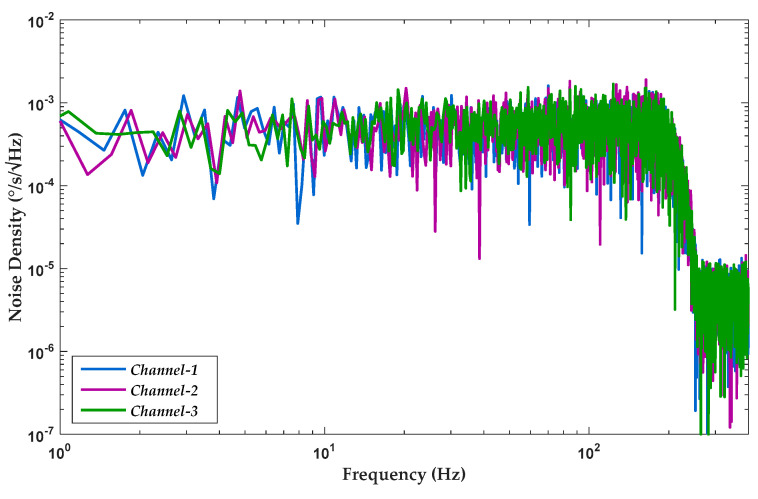
Measured noise floor of the proposed gyroscope ASIC.

The noise floors of triple axes in the proposed gyroscope are shown in Figure 20, demonstrating that in-band noise levels in three channels were nearly consistent. According to noise analysis, average noise density is achieved below 1 m°/s/Hz. These potential sources of output noises usually include noises deriving from references and bias voltages, charge leakage, and white and flicker noise components of analog front-end circuit modules [26]. Besides, in-band output noises keep relatively constant, which also proves the validity of the 100-Hz input bandwidth.

**Figure 21:**
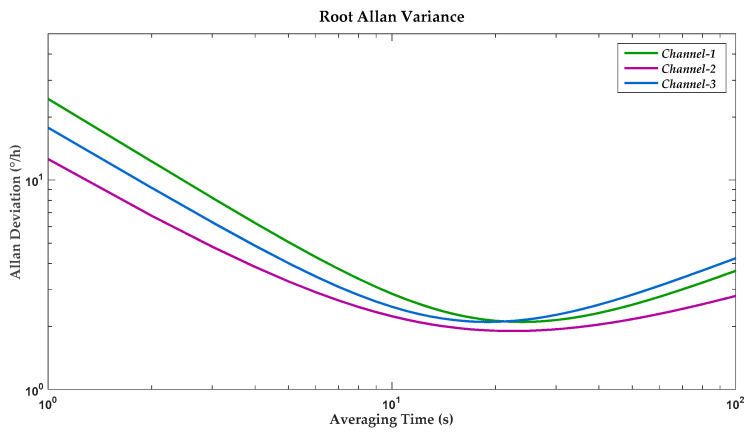
Measured root Allan variance of triple channels at room temperature.

The prototype gyroscope was tested on a settled rate table in a thermostat for exclusion of environmental disturbance on comprehensive performances. Under the condition of preset environmental temperature and humidity, all output measurement data were collected by logic analyzer Agilent 16,804 A and averaged before further data analysis. Bias instability of the overall system was analyzed by calculating standard root Allan variance. Figure 21 illustrates the root Allan variance plot of triple axes for the proposed gyroscope. The calculation results showed a maximum bias instability of 2.1°/h as a representation of the minimum performance among triple sensitive channels.

Table 2 compares this work with state-of-the-art gyroscopes with different systemic architectures and corresponding implementation. The proposed ASIC in this work comprised whole functional modules in gyroscope interface circuits realized by both analog and digital integration method according to specific performance features, as described above. Besides, sensing capacity of triple-axis input angular velocity was achieved with the method of mixed-signal interface integrated circuits. The employed technique of multiplexing design avoided circuit device redundancies and waste and, thus, effectively improved device availability. The obtained gyroscope performances were comparable with other works (in Table 2), especially in the aspect of output nonlinearity.

## 6. Discussions

It is generally acknowledged that micromechanical sensors are advantageous in high integration. This is especially significant in some applications where assembly space is limited. Compared with designs employing interface circuits implemented by discrete circuit elements, the interface ASIC proposed in this work was more flexible in dedicated design flows and was more efficient in power and area consumption. Additionally, the employed implementation of digital signal processing and digital output form enabled the proposed MEMS gyroscope to gain satisfactory compatibility with mainstream industrial and servo controlling systems, which mainly perform in digital domains. Improved efficiency of circuit elements with the aid of time multiplexing design was another characteristic of this work, which avoided redundant circuit modules in repetitive use of single-axis interfaces. In addition to a decrease in chip area, partial power dissipation was also saved. Since the currently designed interface ASIC is implemented by biplate chips, further improvement on circuit integration level is quite significant. Besides, a proven technique of electronic compensation in single-axis gyroscopes is also beneficial to performance improvement in triple-axis angular velocity sensing. Therefore, further work is focused on integration improvement and on-chip error calibration to achieve integrated applications and higher sensing accuracy.

As regards to detailed performances of the implemented overall prototype triple-axis MEMS gyroscope, corresponding interface ASIC dissipated 7.2 mW including analog and digital circuit modules. An effective dynamic range of ±200°/s was achieved within a 100-Hz bandwidth, which satisfies most military and civilian applications. At present, detection accuracy of triple-axis gyroscopes is universally inferior to that of single-axis gyroscopes. Despite there still being room for further optimization, the implemented gyroscope achieved a relatively satisfactory bias instability compared with other works. Besides, the achieved output nonlinearity of 0.012% was restricted in a rather low level, which improves practical applications. In general, balanced performance indexes make the proposed interface ASIC one of the impressive designs among published works.

## 7. Conclusions

This paper presents a triple-axis digital interface ASIC for MEMS vibratory gyroscopes in spatial navigation. A multiplexed incremental zoom ADC was employed in both drive and sense loops and avoided redundantly repetitive consumption of repetitive controlling and resolving logics. The provided effectiveness in device utilization and power dissipation was fully investigated and was also a key feature in this work. All signal controlling and processing algorithms were implemented in digital logics based on precise data conversion in preceding multi-channel ADC. In the aspect of electrostatic driving, a ΣΔ DAC terminated a closed drive loop and gave feedback of sinusoidal voltages to corresponding mechanical electrodes. This work employed nonlinear gain adjustment to avoid introducing frequency distortion, phase noises, and consequent performance deterioration. As a result of flexible drive configuration, the rapid oscillation start-up within 150 ms helped prompt the response ability of overall triple-axis gyroscope. Natural digital outputs of the lowpass filter were compatible with recent mainstream integrated application systems. The prototype interface ASIC was designed and fabricated in a standard 0.35-μm CMOS process. Experimental results showed a bias instability of 2.1°/h.

## Figures and Tables

**Table 1:** Mechanical properties of sense elements.

Mechanical Parameters	Parameter Value
Resonance frequency of drive mode (Hz)	3535
Resonance frequency of sense mode (Hz)	3554
Drive-mode quality factor	32,320
Sense-mode quality factor	34,195
Mechanical bandwidth (Hz)	19

**Table 2:** Summary of published state-of-the-art MEMS gyroscopes.

Reference	[26]	[27]	[28]	[39]	This Work
Year	2011	2016	2017	2020	2019
Interface type	analog	analog + FPGA	mixed-signal	lock-in amplifier	mixed-signal
Power (mW)	6.6	27.28	21	-	7.2
Chip area (mm^2^)	4.3	2.3	7.3	-	9.3
Degrees of freedom	2	3	2	3	3
Dynamic range (°/s)	±1000	±100	500	-	±200
Bandwidth (Hz)	160	80	480	-	100
Nonlinearity (%)	0.5	0.05	-	-	0.012
Bias instability (°/h)	22	1.2	5.5	11.32	2.1
